# Structural analyses of *Arabidopsis thaliana* legumain γ reveal differential recognition and processing of proteolysis and ligation substrates

**DOI:** 10.1074/jbc.M117.817031

**Published:** 2018-04-08

**Authors:** Florian B. Zauner, Brigitta Elsässer, Elfriede Dall, Chiara Cabrele, Hans Brandstetter

**Affiliations:** From the Department of Biosciences, University of Salzburg, Salzburg 5020, Austria

**Keywords:** chemical biology, computational biology, crystal structure, cysteine protease, peptide biosynthesis, pH regulation, plant biochemistry, structural biology, transpeptidation, water displacement model

## Abstract

Legumain is a dual-function protease–peptide ligase whose activities are of great interest to researchers studying plant physiology and to biotechnological applications. However, the molecular mechanisms determining the specificities for proteolysis and ligation are unclear because structural information on the substrate recognition by a fully activated plant legumain is unavailable. Here, we present the X-ray structure of *Arabidopsis thaliana* legumain isoform γ (AtLEGγ) in complex with the covalent peptidic Ac-YVAD chloromethyl ketone (CMK) inhibitor targeting the catalytic cysteine. Mapping of the specificity pockets preceding the substrate-cleavage site explained the known substrate preference. The comparison of inhibited and free AtLEGγ structures disclosed a substrate-induced disorder–order transition with synergistic rearrangements in the substrate-recognition sites. Docking and *in vitro* studies with an AtLEGγ ligase substrate, sunflower trypsin inhibitor (SFTI), revealed a canonical, protease substrate–like binding to the active site–binding pockets preceding and following the cleavage site. We found the interaction of the second residue after the scissile bond, P2′–S2′, to be critical for deciding on proteolysis *versus* cyclization. *cis-trans*-Isomerization of the cyclic peptide product triggered its release from the AtLEGγ active site and prevented inadvertent cleavage. The presented integrative mechanisms of proteolysis and ligation (transpeptidation) explain the interdependence of legumain and its preferred substrates and provide a rational framework for engineering optimized proteases, ligases, and substrates.

## Introduction

Over the last 20 years, plant legumains attracted increasing attention largely due to their dual protease–peptide ligase function (1–4). Contrasting mammals, plants contain multiple legumain isoforms (5, 6). *Arabidopsis thaliana* encodes four legumain forms, two vegetative-type (AtLEGα and γ), one seed-type (AtLEGβ), and a separate grouped (AtLEGδ). The vegetative-type legumains, like AtLEGγ, are involved in plant-programmed cell death (7, 8). This function is especially interesting because plants lack caspases, which are homologous to legumain and serve as key enzymes in mammalian-programmed cell death (9). Several studies showed that plant legumains and caspases share the same substrates and inhibitors due to their preference for acidic sequences such as Tyr-Val-Ala-Asp, Val-Glu-Ile-Asp, and Ile-Glu-Thr-Asp (7, 10, 11). Plant legumain mostly locate to the vacuoles and are, therefore, alternatively referred to as vacuolar-processing enzymes (or VPEs) (12). Legumains are synthesized as inactive precursors, or zymogens, with a tripartite domain organization. It comprises an N-terminal asparaginyl endopeptidase domain (AEP),^2^ an intermediate activation peptide that blocks access to the active site and thus confers enzymatic latency to the zymogen, and a C-terminal legumain stabilization and activity modulation (LSAM) domain, which renders legumain stable at neutral pH and restricts substrate access to the active site (13).

Specific legumain isoforms differ strongly in their peptidase and ligase activities toward certain substrates. For example, of five tested legumains from *Helianthus annus*, *A. thaliana* isoform β, *Ricinus communis* (castor bean), *Canavalia ensiformis* (jack bean) legumain, and *Clitoria ternatea* (butelase-1), only the latter two showed significant ligase activity, whereas the others exhibited only proteolytic activity (14, 15). Recently, it has been shown that AtLEGγ is able to efficiently ligate linear peptides (16). Ligation was also reported for legumains from another kingdom of life, in human and mouse legumain (3, 17, 18). Ligations are especially interesting when peptides are head-to-tail cyclized, thereby producing a large variety of cyclic peptides. Examples are the potent sunflower trypsin inhibitor (SFTI), one of the shortest cyclic peptides, and kalata B1, a member of the so-called cyclotides (14, 15, 19). SFTI serves as an ideal model peptide to study cyclization. Due to their special structural properties such cyclic peptides play important roles in plant defense strategies like pesticidial, insecticidal, antimicrobial, or nematodical activities (20–23). They all share common characteristics like a high thermal, pH, or proteolytic resistance, making them attractive drug scaffolds (23–26).

*In vivo*, precursors of cyclic peptides, like PawS1 of SFTI (26), are ribosomally synthesized and post-translationally modified, *e.g.* by the formation of disulfide bridges or the removal of signal- or propeptides (27). During its maturation, pro-SFTI is processed twice by legumain. Initially, legumain cleaves and releases a flexible N-terminal propeptide from pro-SFTI (19, 27, 28). The subsequent cleavage and release of a C-terminal propeptide is accompanied by a head-to-tail ligation, *i.e.* cyclization, also catalyzed by legumain (19). However, not all peptides are efficiently ligated/cyclized during the second processing step by plant legumain (2, 15). Quite apparently, the peptide sequence and structure determine its preference for cleavage or cyclization/ligation with a strong preference for hydrophobic residues in the so-called P2′ position, which is the second residue after the cleavage site (4, 14, 15, 29, 30). For a definition of the nomenclature of the substrate recognition sites according to Schechter and Berger, please see Ref. 31. However, the specific role of this highly conserved residue remained unclear.

Similarly, the detailed reaction mechanism underlying the plant legumain-mediated ligation reaction remains controversial. For several plant legumain isoforms, a thioester with the catalytic cysteine was postulated as a critical reaction intermediate (32). This so-called enzyme-acyl complex can either be released by a water molecule (*i.e.* hydrolysis, classic proteolytic cleavage) or by the nucleophile of an incoming N terminus. In the latter case, a ligated (or cyclized) peptide product is released from the legumain active site (1, 14, 19). Remarkably, for human legumain ligation was reported to occur at least partly independent of the catalytic cysteine. Indeed, ligation was enhanced if the catalytic cysteine was blocked, presumably by preventing re-hydrolysis of the ligated peptide bond. Lacking the thioester activation, an alternative activation by the proximal aspartimide (succinimide) was suggested (3, 17).

The incomplete atomistic understanding of mechanisms and specificities for proteolysis and ligation by legumain also reflects the lack of crystal structure information on the substrate recognition by a fully activated plant legumain, *i.e.* the catalytic AEP where the C-terminal activation peptide and LSAM domain are released. Here, we report the crystal structure of the peptidase form (AEP) of AtLEGγ in covalent complex with the substrate analogue Ac-YVAD chloromethyl ketone (CMK). The structure maps the important substrate recognition sites before and after the scissile peptide bond, which are referred to as nonprimed and primed recognition sites (31). Biochemical and computational analyses indicated the importance of *cis-trans-*isomerization of the ligation product as well as the shielding from the catalytic water molecule.

## Results

### Overall structure

**Figure 1.**
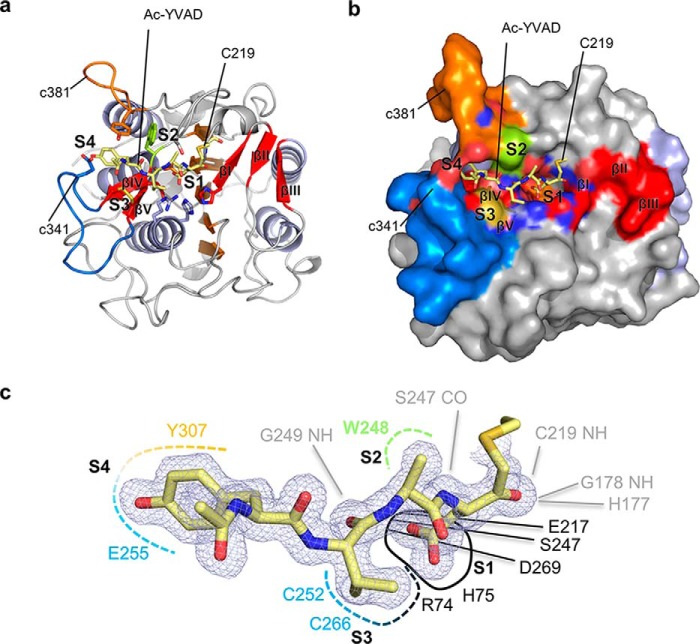
**Interaction topology of the peptidic Ac-YVAD-CMK inhibitor with fully activated AtLEGγ (AEP).** The active form of AtLEGγ is displayed in standard orientation, *i.e.* with substrate running from left to right. The specificity loops c341 and c381 are colored in *blue* or *orange*, respectively. *a*, complex structure of the covalently bound Ac-YVAD-CMK-based inhibitor in a cartoon model. *b,* surface representation of *a* showing how the specificity loops embrace the substrate. *c*, mapping of the substrate recognition sites. The ligand and its electron density (2*F_o_* − *F_c_* map, 1σ contouring) are shown in *blue*. The pockets and its main interactors are indicated with color code: *black*, S1; *green*, S2; *blue*, S3; *orange*, S4. The backbone interactions are shown in *dashed lines*.

For a detailed understanding of how fully activated *A. thaliana* legumain γ (AtLEGγ) recognizes a substrate, we solved the crystal structure of the AtLEGγ peptidase (AEP) in complex with the covalent peptidic CMK-based inhibitor Ac-YVAD-CMK to 1.5-Å resolution (Fig. 1, Fig. S1). For statistics on data collection and refinement see Table 1. The peptidase (AEP) exhibited the characteristic six-stranded central β-sheet (β1–β6), which was surrounded by five major and five minor α-helices (Figs. S1 and S2) (13).

**Table 1:** **Statistics for AtLEGγ-CD in covalent complex with Ac-YVAD-CMK**

	AtLEGγ in covalent complex with Ac-YVAD-CMK
**Data collection**	
Space group	P12_1_1
Cell dimensions	
*a*, *b*, *c* (Å)	41.74, 78.07, 77.72
α, β, γ (°)	90, 92.89, 90
Resolution (Å)	24.56 (1.5)
*R*_merge_	0.05 (0.24)
*R*_meas_	0.07 (0.34)
*R*_pim_	0.05 (0.24)
Total number of observations	161,913 (7,215)
Total number unique	74,201 (3,597)
Completeness	93.5 (91.7)
Multiplicity	2.2 (2.0)
*I*/σ|	8.3 (2.7)
CC1/2	0.99 (0.78)
Wilson B-factor (Å^2^)	10.9

**Refinement**	
Resolution (Å)	24.5–1.50
No. reflections	74,167
*R*_free_/*R*_work_	0.181/0.151
No. atoms	5,178
Protein	4,486
Ligand/ion	22
Water	670
B-factors	16.2
Protein	14.5
Ligand/ion	11.6
Water	27.9
Root mean square deviations	
Bond lengths (Å)	0.006
Bond angles (°)	1.133
Clashcore	1.37
Ramachandran outlier (%)	0.00
Rotamer outlier (%)	0.41

### Delineating the substrate-recognition sites

Especially interesting were the substrate-recognition sites. The nonprimed substrate recognition, *i.e.* the substrate binding preceding the substrate's scissile peptide bond, is facilitated by the edge strands βIV and βV and a plant-specific insertion of 7 amino acids (aa) in the so-called c341 loop (13, 18) as compared with human legumain (Fig. 1, Fig. S1 and S2; c341 and c381 referring to caspase 1 numbering (13)). The c381 specificity-loop, which features a 7-aa insertion compared with mammalian legumain (13) (Fig. S1), also significantly contributed to the nonprimed substrate interaction. Assuming an extended binding mode of the peptide substrate, the primed sites C-terminal to the scissile bond are located on the antiparallel βI–βIII-sheet (Fig. 1, Fig. S2).

### Disorder–order transition upon zymogen activation

**Figure 2.**
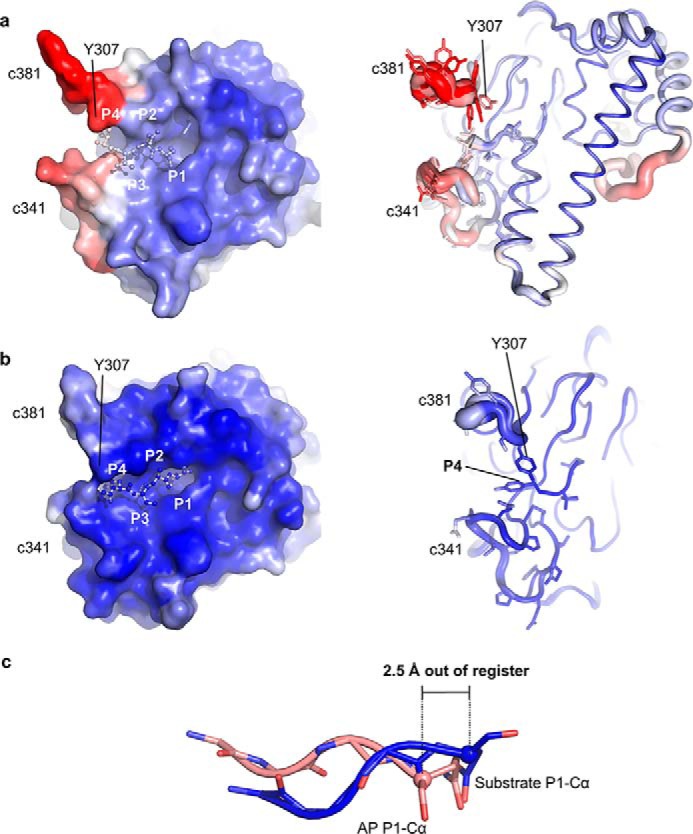
**Substrate recognition in the fully activated AtLEGγ (AEP) form differs from the activation peptide (AP) binding in the zymogenic two-chain state with respect to both structure and dynamics.** Surface (*left*) and cartoon representations are used to color code the conformational variabilities by their crystallographic temperature factors. The color spectrum from *blue* to *red* represents low to high conformational variability, *i.e.* rigid (*blue*) to flexible (*red*). *a,* note the high temperature factors and multiple conformations of the specificity loops c341 and c381 in the zymogenic two-chain state. *b,* the fully activated catalytic domain shows well-ordered specificity loops. *c,* structural superposition of the activation peptide of the zymogenic two-chain state (AP) in *orange* and the substrate Ac-YVAD in *blue*. Note the shift in register of around 2.5 Å at the P1 Cα positions.

When we analyzed the AtLEGγ structure in complex with the Ac-YVAD-CMK ligand we found the specificity loops (c341 and c381) and the edge strand (βIV) highly ordered, contrasting the zymogenic structure, displayed in relative B-factors, which indicate the local flexibility (Fig. 2, *a* and *b*) (16). Although the observed flexibility might be influenced by the packing within the crystal lattice, the observed difference was corroborated by two independent molecules in the asymmetric unit for both the active peptidase and the zymogenic structures, minimizing potential influences by crystal lattice contacts (Fig. 2, *a* and *b*). For Tyr^307^, the change was drastic and particularly functionally relevant, because it defines the S4 substrate–binding site (Figs. 1 and 2).

Notably, the main chain interaction of the peptidic substrate (Ac-YVAD-CMK) with the peptidase differed from that previously found for the activation peptide in the zymogenic structure (Fig. 2) (16). In the Ac-YVAD-CMK substrate analogue, there were two major hydrogen bonds between the carbonyl oxygen of Ser^247^ and amide nitrogen of Gly^249^ to P1 amide nitrogen and P2 carbonyl oxygen, respectively. The P2 carbonyl oxygen was further anchored by the side chain of Arg^74^. By contrast, the activation peptide in the two-chain structure was out of register and shifted for 2.5 Å to the N-terminal direction (Fig. 2*c*). This observed shift is critical in rationalizing how the activation peptide can confer enzymatic latency in the zymogen structure: the out-of-register binding, albeit approximately substrate-like, renders the activation peptide encounter complex unproductive and prevents autocleavage of the activation peptide. The out-of-register shift of the activation peptide as compared with a productive peptide binding is mostly caused by Gln^354^ rather than the classical Asn (or Asp) in the P1 position, preceding the scissile peptide bond. The additional CH_2_ group in the Gln side chain displaces its main chain as well as the neighboring P2 residue for ∼3.8 Å as compared with the Ac-YVAD-CMK. Conversely, the lack of the canonical substrate interactions resulted in the observed flexibility of the prominent c341 and c381 specificity loops in the zymogenic structure, whereas these loops are highly ordered in the substrate-bound state (Fig. 2, *a* and *b*).

### Specificity pockets and active-site elements

The covalently bound Ac-YVAD-CMK substrate was clearly visible in the electron density and allowed for an accurate assignment of the nonprimed specificity pockets (Fig. 1, Fig. S2*b*). The oxyanion hole was formed by the amide nitrogens of Cys^219^ and Gly^178^ as well as of imidazole ring of His^177^ (Nδ1) (Fig. 1, Fig. S2*b*). Similarly as reported for mammalian legumain (13, 18), P1 Asp substrates are best accepted at pH 4.0 (3, 13, 33), where the P1 Asp is protonated within the S1-pocket. The protonated Asp^P1^ carboxylate group was coordinated by Asp^269^ and Glu^217^ at the bottom, Ser^247^ on the upper side (“north”) and Arg^74^ and His^75^ on the lower side (“south”) of the S1 pocket. The P2 Ala interacted hydrophobically with Trp^248^. The P3 Val was constrained by the Cys^252^–Cys^266^ disulfide bridge and the guanidium group of Arg^74^. The P4 Tyr was surrounded by the two prominent c341 and c381 specificity loops with their central residues Tyr^307^ (c381) and the aliphatic part of Glu^255^ (c341). We could further identify a potential site for the catalytic water in perfect position to attack a thioester intermediate. The water was coordinated by the catalytic His^177^ in proximity to the scissile carbonyl of Asp^P1^ (Fig. S3, Fig. 6).

### Cyclization of SFTI by AtLEGγ

**Figure 3.**
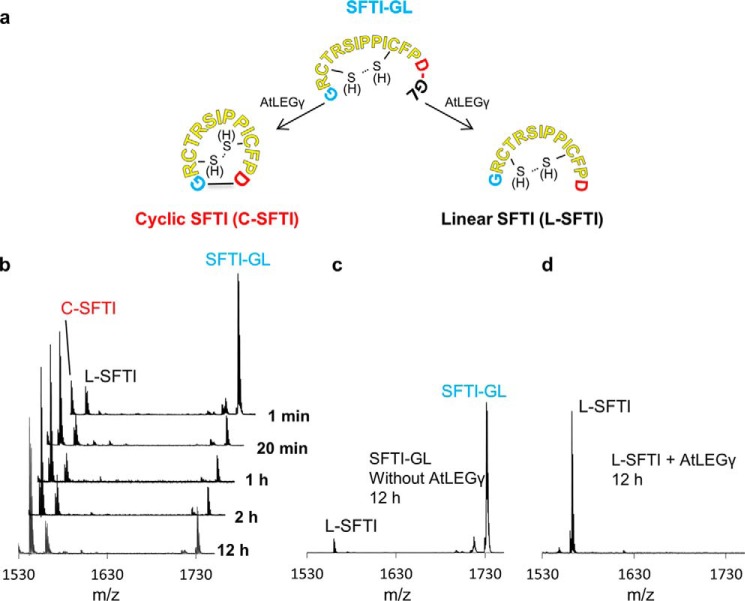
**AtLEGγ efficiently cyclizes SFTI-GL but not cleaved SFTI.** *a,* basic experiment design at pH 6.5. *b*, time course cyclization of SFTI-GL (reduced and oxidized) by AtLEGγ and control experiments. No cyclic SFTI was observed when the SFTI-GL was incubated without AtLEGγ. *c* or *d,* when L-SFTI was incubated with AtLEGγ.

To test whether AtLEGγ can cyclize a modified sunflower trypsin inhibitor precursor peptide (SFTI-GL; ^1^GRCTRSIPPICFPDGL^16^), we monitored time-resolved ligation as catalyzed by activated AtLEGγ. SFTI-GL was cyclized to C-SFTI remarkably fast. Already after 1 min we detected ∼1/3 of the precursor (SFTI-GL) being cyclized (C-SFTI) (Fig. 3). After 20 min, conversion of SFTI to its cyclic form was complete, with ∼10% each resulting in the linear form (L-SFTI) or not being processed at all (precursor SFTI-GL). This distribution and the absolute amounts remained constant for the tested time interval of 12 h, implying and reflecting the proteolytic resistance of cyclic SFTI (Fig. 3). We observed cyclization only in the presence of AtLEGγ and if the precursor SFTI carried the primed residues (*i.e.* the C-terminal Gly^15^–Leu^16^), which were cleaved off by AtLEGγ (Fig. 3, *c* and *d*). Interestingly, we did not find a significant preference for oxidized or reduced SFTI-GL, in agreement with previous reports (15).

### Docking of SFTI reveals a canonical substrate-binding mode

**Figure 4.**
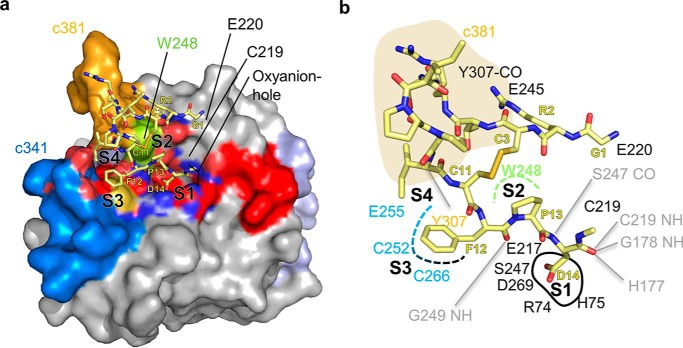
**Docking reveals canonical binding mode of SFTI.** *a,* top view on the docked complex. *b,* interaction map of docked SFTI. *Gray lines* indicate backbone interactions.

To understand how the precursor of SFTI is recognized by AtLEGγ, we performed docking studies guided by the present AtLEGγ-substrate complex structure. The nonprimed substrate-binding sites (S4 to S1) of AtLEGγ served as receptor sites and Asp^14^ of SFTI as the P1 ligand residue (*cf*. Fig. 1). The docking hits with the lowest free energy of binding were in agreement with a canonical binding and resembled the experimentally determined substrate-binding mode (Figs. 1 and 4).

Specifically, we found the carbonyl of P1 Asp^14^ to be docked into the oxyanion hole (formed by the amides of Cys^219^ and Gly^178^ as well as by His^177^) and further backbone interactions such as the amide of Asp^14^(SFTI) with the carbonyl oxygen of Ser^247^ and the carbonyl oxygen of Phe^12^(SFTI) with the amide of Gly^249^, all consistent with the experimentally determined substrate-binding mode (Fig. 1). Furthermore, Pro^13^(SFTI) and Phe^12^(SFTI) bound to the S2 and S3 pockets, respectively. Due to the intramolecular disulfide of Cys^11^(SFTI) with Cys^3^(SFTI), Ile^10^(SFTI) occupied the S4 pocket, interacting with Trp^248^. Interestingly, the docking program positioned the free N terminus of Gly^1^(SFTI) to form an ionic interaction with Glu^220^ close to the catalytic cysteine Cys^219^.

### Proline 13(SFTI) switch allows canonical binding of linear substrate and release of the cyclic product

Careful inspection of the docked structures revealed a major difference of the docked linear SFTI to a cyclic SFTI at Pro^13^(SFTI), which was ∼180° switched (*cis-trans* isomerized) around the Phe^12^–Pro^13^ peptide bond (Fig. S4). This conformational isomerization might be triggered either: 1) by “pulling” Phe^12^(SFTI) to the canonical S3 backbone interaction or 2) by “pushing” SFTI away from AtLEGγ to avoid steric clashes with AtLEGγ; or a combination of both.

Importantly, and contrasting the cyclic SFTI structure (21), the ensemble of NMR solution structures (PDB entry 2AB9) revealed Pro^13^(SFTI) as a wide spectrum of conformations in the SFTI precursor, as did the C-terminal extension, which is cleaved off before cyclization by legumain (2, 14, 28) (Fig. S5). Accordingly, cyclization of SFTI is accompanied by the selection of a Pro^13^(SFTI) conformation (21), which is unfavorable for binding to AtLEGγ. To further substantiate this conclusion, we computationally enforced Pro^13^(SFTI) within the cyclic SFTI to canonically interact with the S2 site, thereby also inducing proper interaction of Asp^14^(SFTI) with the S1 pocket and the oxyanion hole. However, upon releasing these restraints, Pro^13^(SFTI) switched back and pulled the Asp^14^ carbonyl out of oxyanion hole. By contrast, the linear SFTI peptide remained canonically bound also in the absence of such restraints.

### Binding model of primed product residues and their role in ligation

**Figure 5.**
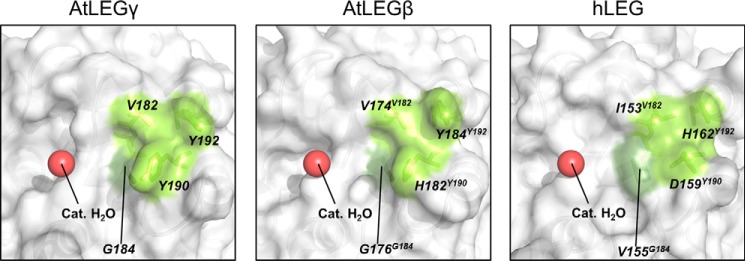
**S2′ pockets for different legumain isoforms and position of the catalytic water.** Different legumain isoforms are displayed in surface representation. *Bright* and *dark green* are the wall- and bottom-forming residues of the different legumain S2′ pockets, respectively. A *red sphere* indicates the position of the catalytic water. The PDB ID of human legumain (hLEG) is 4AWA. The structure of AtLEGβ has been built as an homology model using AtLEGγ as a starting model. The homology model of the catalytic domain of AtLEGβ was built using the software Phyre2 (54).

We next asked how primed residues C-terminal to the scissile peptide bond would bind to AtLEGγ, and to which extent they can prevent the catalytic water from premature hydrolysis of the thioester bond. Thereby, we focused on the P1′–S1′ and P2′–S2′ interactions, because these are reported to be especially important for ligation (2, 4, 15, 34) and, due to the known constraint of the P1–S1 interaction, can be reliably extrapolated. For stereochemical reasons the P1′ residue must have the side chain exposed near the catalytic cysteine Cys^219^ and Glu^220^, which delineate the S1′ pocket. We further found a remarkably pronounced S2′ pocket in AtLEGγ, which is bordered by Val^182^ (human Leg-Ile^153^; h-Ile^153^), Tyr^192^ (h-His^162^), Tyr^190^ (h-Asp^159^), and Gly^184^ (h-Val^155^), with Gly^184^ forming the bottom of the S2′ pocket (Fig. 5). The deep S2′ pocket resulted, among others, from a conserved, plant-specific insertion at position 190 (Tyr^190^ in AtLEGγ or butelase-1; Fig. S1). Furthermore, the S2′ pocket is deepened by the basement residue Gly^184^ as compared with the more bulky Val^150^ in human legumain (Fig. 5, Fig. S1). To explore the binding mode of a dipeptide at the S1′ and S2′ sites, we modeled a C-terminal extension of the docked SFTI to obtain initial positions of the P1′ and P2′ residues.

**Figure 6.**
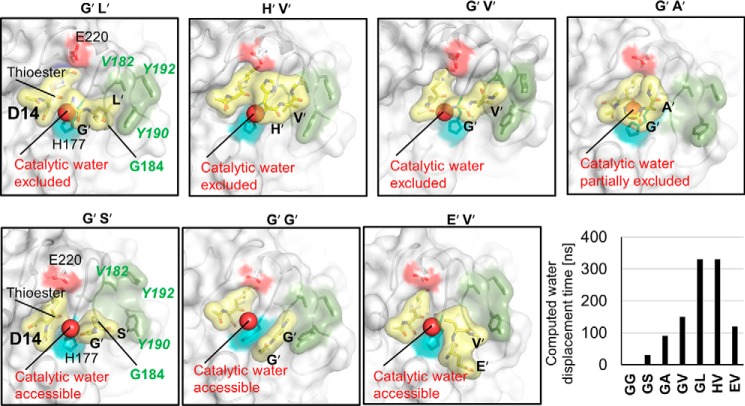
**S2′ pocket of plant legumains has high affinity for hydrophobic P2′ residues, critical for water displacement.** All structures are results of 330-ns molecular dynamics simulations with identical starting structures, despite the indicated mutation of the primed residues. The different primed tails are labeled *above* each structure. *Yellow,* representation of the still bound product, which is thioester-bound to Cys^219^ via Asp^14^; the released primed peptides at the end of the simulation are also indicated. *Cyan,* catalytic histidine. *Red,* Glu^220^. *Dark green*, S2′ pocket. The putative catalytic water is highlighted. The individual computed water exclusion times are also shown.

To further study the ligation/transpeptidation, we assumed a thioester intermediate between the catalytic cysteine and the P1 residue. Using molecular dynamics studies we tested the retention time of the cleaved dipeptides (*i.e.* P1′–P2′) for sequences that have been reported in efficient ligation/cyclization substrates, *e.g.* Gly^15^–Leu^16^(SFTI) or His^15^–Val^16^(SFTI) (15). Remarkably, these two dipeptides remained bound within the pocket even after 330-ns molecular dynamics simulation and displaced the putative catalytic water (Fig. 6). By contrast, dipeptides like Gly^15^–Gly^16^(SFTI) or Gly^15^–Ser^16^(SFTI) rapidly drifted out of the S1′–S2′ pockets during molecular dynamics simulations, with retention times <30 ns. Subsequently, the catalytic water entered the active site nearby His^177^, ready to hydrolyze of the thioester bond (Fig. 6). The dipeptides Gly^15^–Ala^16^(SFTI), Gly^15^–Val^16^(SFTI), or Glu^15^–Val^16^(SFTI) competed with water for ∼100–150 ns, consistent with the intermediate hydrophobicity of Gly^15^–Ala^16^(SFTI) or Gly^15^–Val^16^(SFTI); the negative charge of Glu^15^ is disfavored as P1′ residue due to its proximity to Glu^220^, which is partially balanced by the hydrophobic P2′ Val^16^ (Fig. 6).

## Discussion

### The binding mode of the activation peptide in the zymogen and a substrate differ markedly

In this study we solved the crystal structure of AtLEGγ in complex with Ac-YVAD-CMK (Fig. 1, Figs. S1 and S2). In this structure the binding mode of the peptidic substrate to the active site markedly differed from that seen for the activation peptide in the zymogen form (Fig. 2). Although the P1 Gln^35^4 of the activation peptide mimics a P1 asparagine in the substrate, it induced a partial frameshift of ∼2.5 Å in the activation peptide backbone. This shift leads to distorted backbone–backbone interactions and translates into more disordered specificity loops (c341, c381; Fig. 1, Fig. S2). By contrast, the canonical binding triggered an ordering of the S3–S4 pockets, resulting in a tight binding of the P3 and P4 residues.

### Structure-derived AtLEGγ specificity profile

The covalently bound Ac-YVAD-CMK allowed to deduce the specificity of the nonprimed recognition sites (Fig. 1). The S1 pocket is bipolar and sterically matches with Asp and Asn, thus explaining its strong preference for Asn and protonated Asp at P1. The open S2 pocket with its hydrophobic basement (Trp^248^) explains the preference for hydrophobic residues. The preference for mixed hydrophobic and partially negative P3 residues is consistent with Arg^74^ and the redox-sensitive disulfide bridge Cys^252^–Cys^266^ of the S3 pocket. The S4 site is very adaptive, reflecting the conformational variability of the specificity conferring c341 and c381 loops (Fig. 2). These structure-derived specificity predictions are in agreement with experimentally determined specificities. For example, the caspase-1 (YVAD) inhibitor was reactive toward AtLEGγ, whereas the caspase-3 inhibitor (DEVD) was not (8, 35). This observation is in agreement with the negatively charged S4 pocket, which should exclude a negatively charged P4 residue. Similarly, the reported autocleavage sites of AtLEGγ, *i.e.* ^340^ADAN or ^350^RVTN, match the structure-derived specificity profile (16).

### SFTI-binding mode mimics the binding mode of the α6 helix in the two-chain form of AtLEGγ

Docking of the SFTI inhibitor to the active site positioned its N terminus Gly^1^(SFTI) next to Glu^220^, close to the catalytic cysteine (Fig. 4). This stand-by position enables a coordinated displacement of the primed SFTI (product) residues Gly^15^–Leu^16^(SFTI).

We have previously shown that AtLEGγ can be activated to a pH-stable intermediate (16). This two-chain form is a noncovalent complex of the catalytic domain and the C-terminal domain comprising the α6 helix and LSAM (legumain stabilization and activity modulation) module. Thereby, the α6-helix was shown to act as critical gatekeeper for ligation substrates, which was proposed to be specifically unlocked by a suitable ligation substrate, whereas preventing premature proteolysis. The N terminus of SFTI exactly coincides with the ionic anchorage site of the α6-helix, *i.e.* Arg^355^ binding with Glu^220^. Thus, SFTI mimics the interaction seen in the α6-helix (Fig. S6). Indeed, we could detect significant cyclization of SFTI-GL by the two-chain form, further supporting the correctness of our docking model (Fig. S6).

### Primed side interaction favors cyclization by preventing pre-mature thioester hydrolysis

Although several reports indicated an essential role of the P1′ and P2′ residues in ligation (4, 14, 15, 29, 30), their mechanistic relevance remained so far unclear. Our analysis identified a prominent hydrophobic S2′ pocket, specific to plants. Efficient ligases such as jack bean legumain, butelase-1, and AtLEGγ all share an aromatic residue (Tyr or Phe) at position 190 and a glycine at position 184 (Fig. 5, Fig. S1) (14, 15, 36).

Our computational studies showed that the catalytic water could be displaced by the presence of the P1′–P2′ dipeptide binding, in a sequence-dependent manner (Fig. 6). Hydrophobic P2′ residues had longer retention times, correlating with experimentally observed preferences in ligation substrates (15).

We should note, however, that a recent publication by Yang and colleagues (37) proposed the primed nucleophilic ligation substrate employs a nonprimed binding site, *i.e.* it binds to the left side rather to the right side as shown in Fig. 6. This conclusion was presumably motivated by the C247A mutant, which strongly enhanced ligase activity. However, this proposition is sterically conflicting with the binding of the nonprimed ligase substrate (Figs. 1*a* and 2, Fig. S2*b*).

To test our catalytic water displacement model, we compared the cyclization efficacy between AtLEGγ and AtLEGβ. The latter has Tyr^190^ (in AtLEGγ) substituted to histidine, thus rendering the S2′ pocket less hydrophobic. Indeed we detected a significant higher portion of cleaved SFTI (linear SFTI) than cyclic (Fig. S7), consistent with earlier reports (14). Conversely, AtLEGβ may be a superior ligase over AtLEGγ for substrates with P2′ residues optimized for AtLEGβ's amphiphilic S2′ site. These findings are in perfect agreement with a computational report on human legumain-mediated transpeptidation, which was only possible if water was excluded from the active site (38). Finally we note that the proposed water displacement model is consistent with the reportedly low proteolytic activity of butelase (15) as well as the here observed ≈5000-fold decreased proteolytic activity of AtLEGγ as compared with human legumain (Fig. S8).

### Model of cyclization

**Figure 7.**
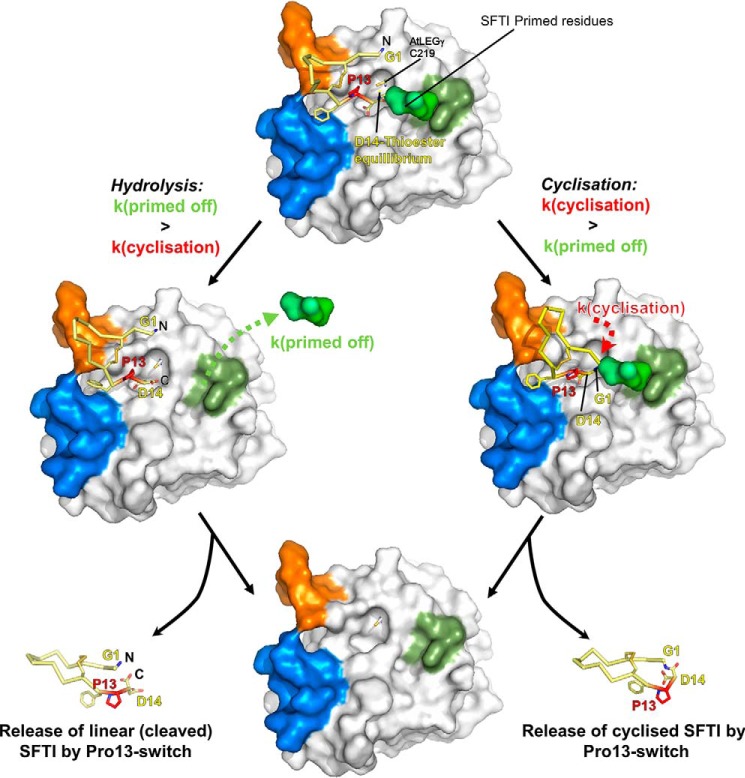
**Water exclusion model of SFTI cyclization and proteolysis.** *Top,* SFTI-GL binds canonically (like a substrate) to AtLEGγ. The primed GL tail occupies the S2′ pocket. The N terminus (Gly^1^) is positioned close to the catalytic cysteine. The attack of the catalytic cysteine creates the acyl-enzyme intermediate, which can be stabilized by the GL-tail shielding it from water. *Left,* cleavage is achieved when the primed tail of SFTI cannot productively interact with the S2′ pocket, which leads to hydrolysis of the thioester, resulting in linear SFTI (L-SFTI). *Right,* the N terminus of SFTI can attack the thioester and cyclic SFTI (C-SFTI) is produced. The Pro^13^ *cis-trans-*isomerization triggers the efficient off-dissociation. *Bottom*, the products of the hydrolysis and the cyclization reaction (shown at the *bottom left* and *bottom right*, respectively) are released from enzyme AtLEGγ (*bottom center*).

Based on our findings, we hypothesize the cyclization of SFTI is performed as illustrated in Fig. 7. Craik and colleagues (2, 14) proposed that pro-SFTI is cleaved and ligated sequentially, whereby the N-terminal segment of pro-SFTI is initially released because of a kinetically preferred asparagine (Asn^1^(SFTI)) cleavage site (Fig. S5) (14, 28). In a second step, the N terminally trimmed SFTI binds canonically with Asp^14^(SFTI) into the active site, primarily exploiting the S4 to S2′ sites, as we observed in our docking studies (Figs. 4 and 6). The catalytic cysteine can then form the acyl-enzyme intermediate, which is long-lived due to the above described water displacement model (Figs. 6 and 7). Subsequently, we propose the nucleophilic Gly^1^(SFTI) to bind to the S1′ site, thereby displacing the primed product residues (2), followed by aminolysis of the thioester resulting in the cyclic peptide.

**Figure 8.**
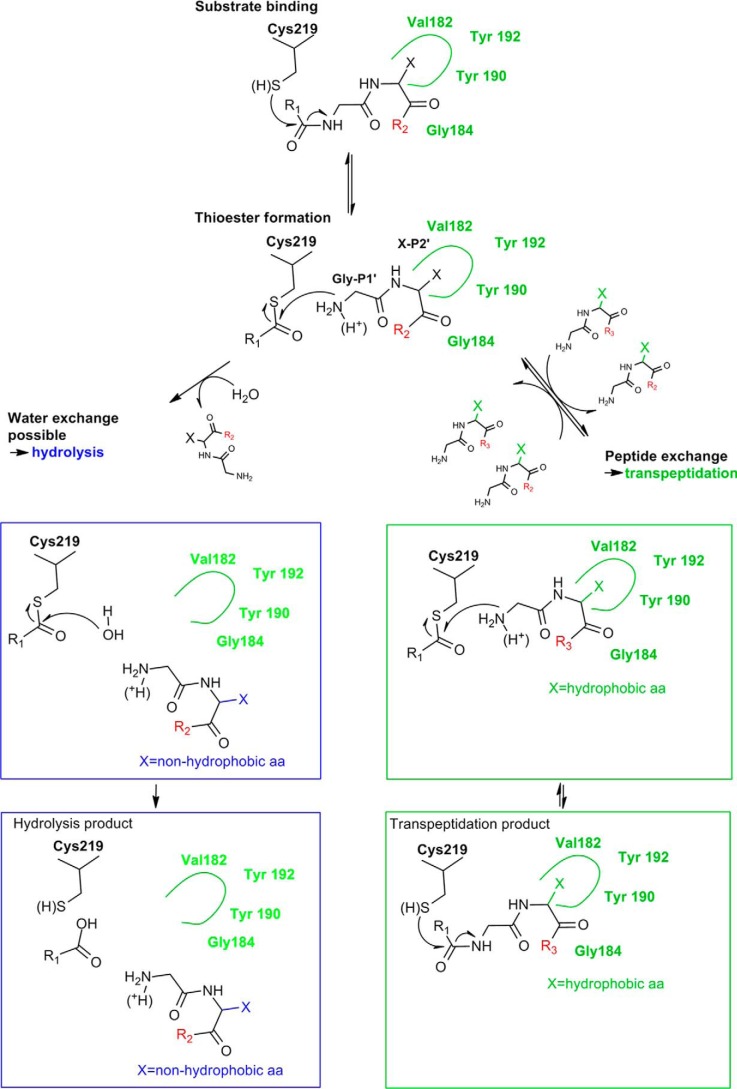
**Mechanistic scheme of transpeptidation and hydrolysis.** *Green rectangles* show transpeptidation reactions induced by a hydrophobic residue in P2′. The hydrolysis reaction pathway is indicated by *cyan rectangles*, induced by a nonhydrophobic residue in P2′. Starting point: *green dashed rectangle*. A substrate (R1 + R2) binds to the active site of AtLEGγ. The attack of Cys^219^ creates a thioester and free N terminus at the primed site. *Left pathway*, if the initially bound substrate (*green dashed line*) carries a nonhydrophobic residue in P2′, the primed product can dissociate after the formation of the thioester and water will exchange. Consequently, this results in hydrolysis of the thioester and the release of the hydrolysis product. *Right pathway*, if P2′ is hydrophobic, the primed site peptide stays bound and prevents the exchange of catalytic water, resulting in an equilibrium between thioester and peptide bond. In the presence of a suitable transpeptidation substrate (R3), an exchange between the initially bound primed product and the transpeptidation peptide can happen. This results in a new equilibrium between thioester and peptide bond, forming the transpeptidation product. The varying protonation state of the released primed N terminus is indicated. Only a deprotonated N terminus is able to attack the thioester, not a protonated one. This relationship explains the pH dependence of transpeptidation, which is more efficient at neutral pH than acidic pH.

A possible reaction scheme is proposed in Fig. 8, which is in agreement with several experimental findings. First, in ligation experiments in the presence of H_2_O^18^ an incorporation of O^18^ into the ligation product could not be observed, indicating that the acyl-enzyme was not H_2_O^18^ hydrolyzed before it was ligated (14). Second, for the homologous caspases, it has been shown that the caspase inhibitor p35 binds the enzyme canonically and thereby displaces the catalytic water. The authors were consequently able to detect a long-lived thioester intermediate in the electron density (39). Third, for the macrocyclase domain of PatG, primed residues need to stay bound after forming the acyl-enzyme intermediate to exclude water from the active site, albeit achieved by different structural principles (40). Upon cyclization Pro^13^(SFTI) *cis-trans*-isomerization is conformationally enforced (Fig. S4) (21, 41), resulting in a decreased affinity and release of the cyclic product (Fig. S4).

Daly *et al.* (41) reported that the P1 Asp^14^ is hydrogen (and ionically) bonded to Arg^2^ in cyclic SFTI, which constraints Pro^13^ in the conformation unfavorable for binding. By contrast, in the D14A SFTI mutant a *cis-trans-*isomerism of Pro^13^ (Pro^13^ switch) was observed. We proposed a similar situation in our D14N SFTI mutant, which should be able to sample more Pro^13^ conformations, leading to re-binding to the active site with the possibility of cyclic SFTI-D14N to be cleaved. This is what we indeed observed, the cyclic SFTI-D14N was a metastable reaction intermediate toward the stable cleaved product (Fig. S9). By combination of high resolution crystallographic studies with computational and biochemical studies we here provide a both detailed and integrative mechanism of peptide bond cleavage and cyclization. The here developed concepts allow to explain and reconcile many published data and to rationally design enzymes and substrates with improved properties in proteolysis and ligation.

## Experimental procedures

*A. thaliana* AEP (legumain) isoform γ (AtLEGγ) full-length clone U10153, locus: AT4G32940, was obtained from TAIR database. Restriction enzymes and T4 ligase were obtained from Fermentas (St. Leon-Rot, Germany) and Pfu Ultra II Fusion HS DNA polymerase was obtained from Stratagene (La Jolla, CA). Custom-made primers were obtained from Eurofins Genomics (München, Germany) and sequence analyses were performed at Eurofins MWG Operon (Martinsried, Germany). *Escherichia coli* strain XL2 Blue (Stratagene) was used for subcloning expression constructs. To produce fully glycosylated protein, the *Leishmania tarentolae* expression system (LEXSY; Jena Bioscience, Germany) was used (42). All reagents used were of the highest standard available from Sigma (München, Germany) or AppliChem (Darmstadt, Germany).

### Cloning

An N-terminal truncated mutant (Ser^56^–Ala^494^) of *A. thaliana* proLEG isoform γ (referred in this work with pro-AtLEGγ) was amplified by PCR (Eppendorf Mastercycler ep gradient thermal cycler) to exclude the N-terminal ER-signal peptide and vacuolar sorting signal (43). *A. thaliana* legumain isoform γ full-length clone U10153 was used as a template. An appropriate forward primer containing an XbaI restriction site, His_6_ tag, and a tobacco etch virus protease-cleavage site, AGCTCTCGAGTCTAGAGCACCACCATCACCACCACGAAAACCTGTATTTTCAGTCCGGTACTAGGTGGGCTGTTCTAGTCGCCG and a reverse primer containing a NotI restriction site, AGCTGCTCAGCGCGGCCGCCTATGCACTGAATCCACGGTTAAGCGAGCTCCAAGGAC, were used. Subsequently, the PCR product was cloned into the pLEXSY-sat2 vector utilizing the XbaI and NotI restriction sites. The expression constructs carried an N-terminal signal sequence for secretory expression in the LEXSY supernatant. Correctness of all constructs was confirmed by DNA sequencing.

### Cell culture, protein expression, and purification

Expression constructs were stably transfected into the LEXSY P10 host strain and grown at 26 °C in BHI medium (Jena Bioscience, Germany) supplemented with 5 μg/ml of heme in 50 units/ml of penicillin and 50 mg/ml of streptomycin (Carl Roth GmbH, Germany). Positive clones were selected by addition of nourseothricin (Jena Bioscience). Protein expression was carried out as described elsewhere (13). Recombinant protein was removed from the LEXSY supernatant via Ni^2+^ purification using nickel-nitrilotriacetic acid Superflow resin (Qiagen, Hilden, Germany). The wash buffer contained 20 mm HEPES, pH 7.2, 300 mm NaCl, and 10% glycerol. The elution buffer was composed of 20 mm HEPES, pH 7.2, 300 mm NaCl, 10% glycerol, 250 mm imidazole, and 0.3 mm *S*-methyl methanethiosulfonate. The elution fractions were concentrated using Amicon® Ultra centrifugal filter units (3-kDa molecular mass cut off, Millipore) and desalted using PD-10 columns (GE Healthcare) to the final buffer: 20 mm HEPES, pH 7.2, 50 mm NaCl.

### Preparative autoactivation to yield two-chain state and protease only

2–3 mg/ml of pro-AtLEGγ were incubated in autoactivation buffer A (100 mm Tris, 100 mm BisTris, 100 mm citrate, pH 4.0, 100 mm NaCl) for 16 h at 30 °C to generate two-chain AtLEGγ. To prepare the protease only, 2–3 mg/ml of pro-AtLEGγ were incubated at 30 °C in autoactivation buffer B (100 mm Tris, 100 mm BisTris, 100 mm citrate, pH 4.0, 100 mm NaCl, and 2 mm DTT) for 2 h. All samples were checked for the presence or absence of the α6-LSAM domain by SDS-PAGE. After autoactivation, two-chain or protease-only samples were subjected to gel filtration chromatography utilizing an Äkta-FPLC system (SEC 200 10/300 GL column, buffer: 20 mm citrate, pH 4.2, 100 mm NaCl) to remove degradation products and DTT. Afterward, the respective fractions were either used directly for enzymatic assays or aliquoted and frozen at −20 °C.

### Protein crystallization

AtLEGγ was purified as described above. Before concentration AtLEGγ was inhibited with Ac-YVAD-CMK at pH 4.0. After inhibition a SEC run was performed (SEC 75, 15 mm citric acid, pH 4.5, 80 mm NaCl) and corresponding fractions were pooled and concentrated to ≈5 mg/ml. Crystallization screening was carried out using the sitting-drop vapor-diffusion method utilizing a Hydra II Plus One (Matrix) liquid-handling system. Crystals grew within 3–6 days in a condition consisting of 4% PEG 4000, 100 mm sodium acetate, pH 4.6.

### Data collection and processing

An X-ray diffraction data set was collected on beamline ID29 at the ESRF at 100 K. The beamline was equipped with a Pilatus6M detector. Data collection was performed using a crystal-to-detector distance of 280.919 mm and a wavelength of 0.976251 Å. The exposure time was 0.04 s at 2.3% transmission. Data processing was performed by using iMOSFLM (53) and Aimless from the CCP4 program suite (44). Packing density was calculated according to Matthews (45). An initial model could be generated by molecular replacement with the two-chain form of AtLEGγ (PDB code 5NIJ), the structure was refined by using Refmac 5 (46) and phenix.refine (47). The structure was deposited with the Protein Data Bank under PDB code 5OBT.

### Synthesis and analytics of SFTI-peptides

#### 

##### Materials

Fmoc (*N*-(9-fluorenyl)methoxycarbonyl)-protected amino acids, Rink-amide MBHA resin (loading 0.45 mmol/g), H-Asp(OtBu)-2-chlorotrityl-resin (loading 0.60 mmol/g), 2-(1*H*-benzotriazole-1-yl)-1,1,3,3-tetramethyluronium hexafluorophosphate (HBTU), *N*,*N*-diisopropylethylamine (DIPEA), piperidine, *N*,*N*-dimethylformamide, *N*-methyl-2-pyrrolidone, dichloromethane, diethyl ether, acetonitrile, and trifluoroacetic acid were purchased from Merck Millipore (Germany), Biosolve (The Netherland), and Iris Biotech (Germany). Triisopropylsilane, 1,2-ethanedithiol, thioanisole, and *N*-hydroxybenzotriazole (HOBt) were purchased from Sigma-Aldrich (Germany). α-Cyano-4-hydroxycinnamic acid was purchased from Acros Organics (Germany).

##### Methods

Solid-phase peptide synthesis was carried out on an automatic peptide synthesizer (Syro I, Biotage). The analytical and semipreparative HPLC equipment was from Thermo Fisher Scientific (model Ultimate 3000). The analytical column was from Thermo Fisher Scientific (Syncronis C-18, 4.6 × 250 mm, 5 μm), the semipreparative column was from Macherey Nagel (NUCLEOSIL® C-18, 250 × 10 mm, 5 μm). MALDI-TOF mass spectra were recorded on an Autoflex mass spectrometer from Bruker Daltonics using α-cyano-4-hydroxycinnamic acid as matrix.

### Peptide cyclization experiments

Cyclization experiments were performed with 500 μm of the corresponding peptide and 0.5 μm AtLEGγ at 30 °C. The reaction buffer consisted of 100 mm MES, pH 6.5, and 100 mm NaCl. The ligation mixture was desalted by using the ZipTip Pipette Tip C-18 (Merck Millipore) and analyzed by MALDI-TOF-MS (Autoflex, Bruker Daltonics, matrix: α-cyano-4-hydroxycinnamic acid).

### Testing peptidase activity

The proteolytic activities of selected activation intermediates and isoforms were measured using 20 μm of the fluorogenic substrate Z-VAN-MCA or IETD-MCA in activity buffer A adjusted to the desired pH value (100 mm Tris, 100 mm BisTris, 100 mm citrate, 100 mm NaCl) at 20 °C. For each measured pH value, the reaction was started by adding around 0.5–2 μl of the respective sample to the premixed 49.5 to 48-μl mixture. The concentration of each enzyme in the assay was <1.5 μm if not otherwise stated. The substrate turnover was measured at an excitation and emission wavelength of 370 and 450 nm, respectively, in an Infinite M200 Plate Reader (Tecan). Proteolytic activity was determined by calculation of the initial slopes of the time-dependent substrate turnover. Each measurement was done in triplicate.

### Structure preparation and docking

Starting from the crystal structure of the fully activated AtLEGγ, first the inhibitor was removed from the system. Afterward, the enzyme was titrated at pH 6.0 (experimental pH) using the Protonate 3D function of MOE2016.08 (48).

The structure of the substrate, SFTI, was retrieved from the Protein Database (49): PDB codes 1JBL (cyclic) and 1JBN (noncyclic) (21). Because these PDB files comprise several structures, only one chain was kept and protonated at pH 6.0 as described above for the enzyme. In case of the noncyclic inhibitor (PDB code 1JBN) the C terminus was appended by NME (*N*-methyl) to maintain neutrality.

The docking simulations were performed using the following settings of the software package of *MOE 2016.08.* In the potential energy setup panel AMBER99 was chosen as force field. As placement protein–protein docking was employed to find the optimal docking hits. Each run was adjusted to pre-placement of 10,000, placement of 500 and refine 30 conformations as a cut-off. The top poses were retained for further analysis, investigating the H-bond distances between the substrate and the enzyme. For AtLEGγ, residues Cys^219^, Gly^187^, His^75^, Arg^74^, Cys^252^, Cys^266^, Asp^217^, Ser^247^, Asp^269^, Trp^248^, Gly^249^, Glu^255^, and Tyr^307^ were defined as the binding pocket. In addition, as docking site of the substrate Asp^14^ was chosen. The best docking hits were optimized using the energy minimization function of MOE2016.08 (48, 50) with AMBER99 force field method. The docking results were judged by proper interactions with the S1 pocket and major backbone interactions. In addition to the well-established computational scoring function, the interaction-based accuracy classification method (51) was used to identify the docking hits, which included an interaction pattern of Asp^14^(SFTI) in the S1 pocket resembling the experimentally determined geometry (Fig. 1).

### Thioester generation and optimization

To generate the tripeptides for the molecular dynamics simulations, first AtLEGγ was superimposed with the crystal structure of the human legumain-cystatin (PDB code 4N6O) complex, because in that complex also primed residues are bound. The P1 to P2′ residues were mutated to the sequence of interest and terminated by ACE (acetyl) and NME (*N*-methyl), respectively. The so generated complex was optimized using the energy minimization function of MOE2016.08 (48) with the AMBER99 force field method. Finally, the peptide bond between the P1–P1′ residues was broken, a covalent bond between the carbonyl carbon of the P1 aspartic acid and SG(Cys^219^) was generated and the complex was reoptimized. For the molecular dynamics studies the P1′ and P2′ residues were systematically mutated using the Protein Builder function of MOE2016.08 and reoptimized (MOE2016.08, AMBER99 force field).

### Molecular dynamics

The protein–peptide complex was solvated in an 80-Å cubic box of waters and counterions (either Na^+^ or Cl^−^) were added to maintain neutrality of the overall protein. Afterward, a series of equilibration steps were carried out by performing molecular dynamics annealing runs for 100 ps at temperatures 50, 150, 200, and 250 K and for 330 ns at 298.15 K (in 11 steps, after each 30 ns the coordinates were saved for further analysis). The molecular dynamics calculations were accomplished using AMBER99 force field as implemented into NWChem 6.6 (52).

## Author contributions

F. B. Z. designed and performed most experiments. F. B. Z., B. E., E. D., H. B. discussed and interpreted all experiments. C. C. synthesized the peptides for ligation, assayed and interpreted the ligation by mass spectrometry. F. B. Z. and H. B. wrote the manuscript, all authors proofread and agreed with the paper.

## Supplementary Material

Supporting Information

## Footnotes

2 The abbreviations used are:

AEP: asparaginyl endopeptidase domain
LSAM: legumain stabilization and activity modulation
SFTI: sunflower trypsin inhibitor
CMK: chloromethyl ketone
AtLEGγ: *A. thaliana* legumain γ
BisTris: 2-[bis(2-hydroxyethyl)amino]-2-(hydroxymethyl)propane-1,3-diol
SEC: size exclusion chromatography
Z: benzyloxycarbonyl
L-SFTI: linear SFTI
MCA: 4-methyl-coumaryl-7-amide.
