# Supplementary material for: Structural analyses of Arabidopsis thaliana legumain γ reveal differential recognition and processing of proteolysis and ligation substrates
Source: J Biol Chem. 2018 Apr 8;293(23):8934–46. doi: 10.1074/jbc.M117.817031 (PMC5995516; doi:10.1074/jbc.M117.817031)
Supplement: Supporting Information [file supp_293_23_8934__index.html]

Structural analyses of Arabidopsis thaliana legumain γ reveal the differential recognition and processing of proteolysis and ligation substrates — Structural analyses of Arabidopsis thaliana legumain γ reveal differential recognition and processing of proteolysis and ligation substrates — Proteolysis and ligation by plant legumain — Supporting Information 

# Structural analyses of *Arabidopsis thaliana* legumain γ reveal differential recognition and processing of proteolysis and ligation substrates

## Supporting Information

- Supplemental data (.pdf, 2.2 MB) - Supplemental data and figures
