# Supplementary material for: Structural analyses of Arabidopsis thaliana legumain γ reveal differential recognition and processing of proteolysis and ligation substrates
Source: J Biol Chem. 2018 Apr 8;293(23):8934–46. doi: 10.1074/jbc.M117.817031 (PMC5995516; doi:10.1074/jbc.M117.817031)
Supplement: Supporting Information [file 10.1074_M117.817031_jbc.M117.817031-1.pdf]

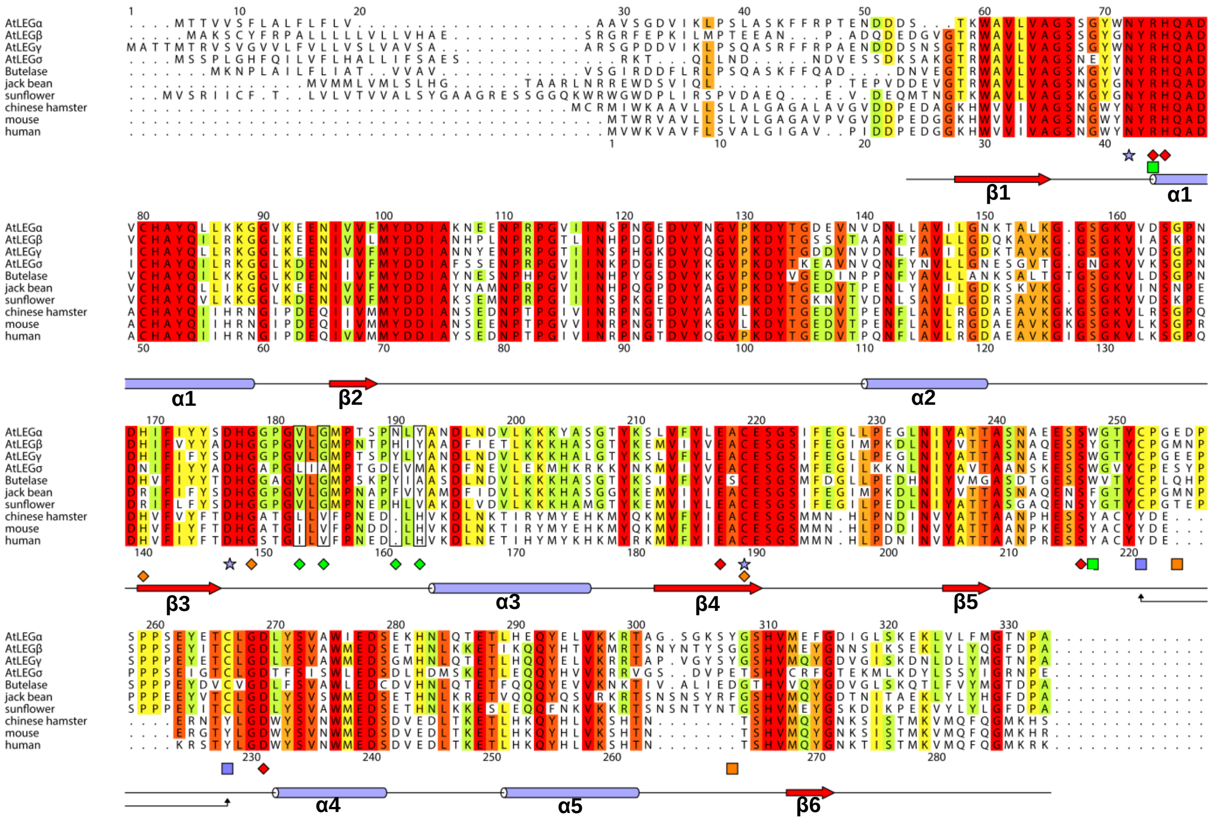

Supplementary Figure 1: Multiple sequence alignment of legumain isoforms.

Aligned and displayed with Clustal W and aline. Upper and lower numbering corresponds to AtLEGγ and human Legumain respectively. The major secondary structure elements are of the AtLEGγ catalytic domain only structure and are depicted below the sequences. Blue star, catalytic residues; red diamonds, S1 residues, green rectangles, S2 residue; blue rectangles, S3 residues; Orange rectangles, S4 residues. The S2‘ residues are highlighted by green diamonds (see boxes).

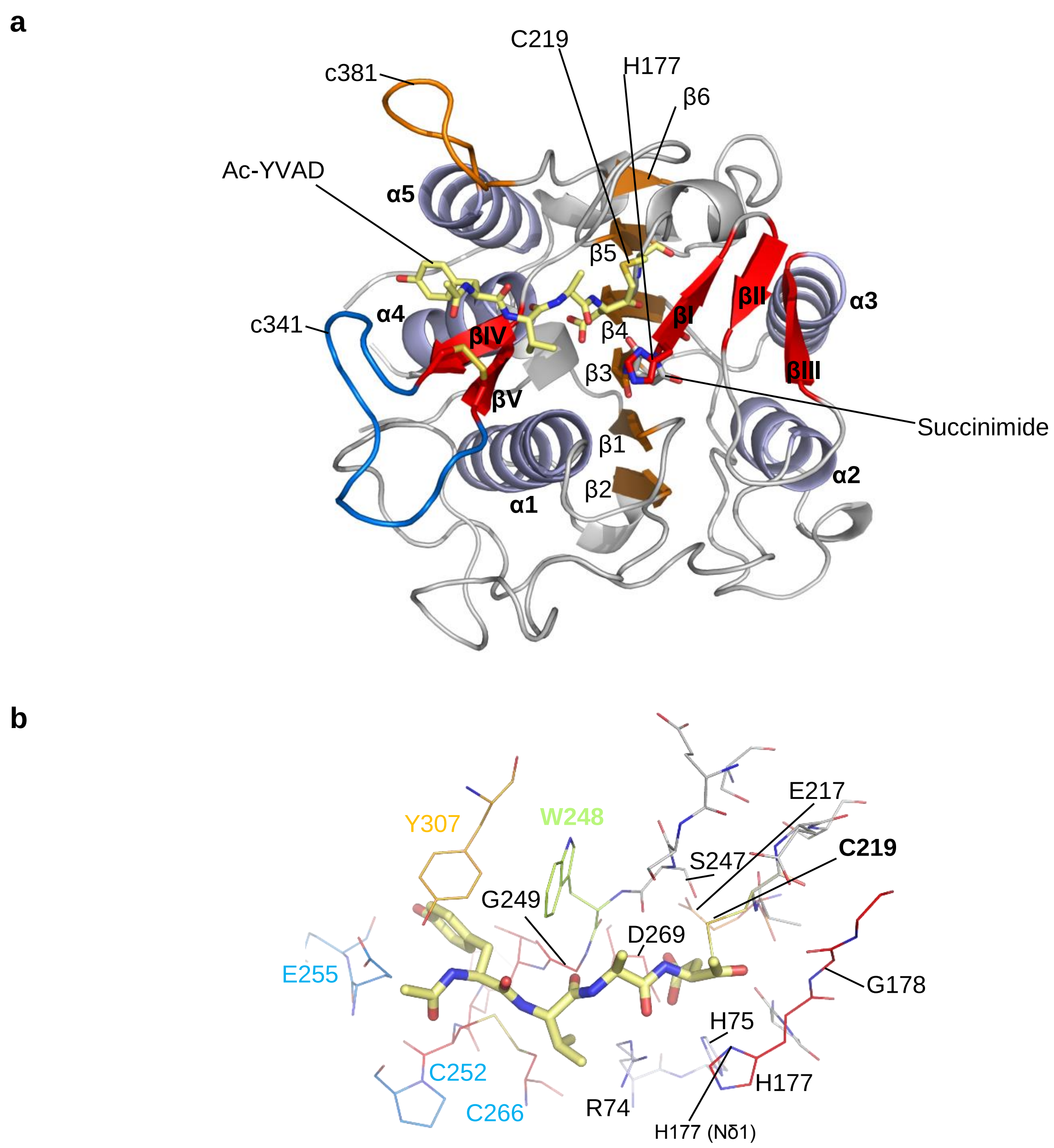

Supplementary Figure 2: Topology of catalytic domain-only and active site. (a) Topology of the catalytic domain only in complex with Ac-YVAD-CMK. The specificity loops and catalytic residues are labelled. Central β-sheet, orange; Major α-helices, blue; substrate recognition is mediated by non-primed (left) and primed β-sheets (βI-βV, right) in red. (b) Active site zoom with relevant substrate interacting residues labeled.

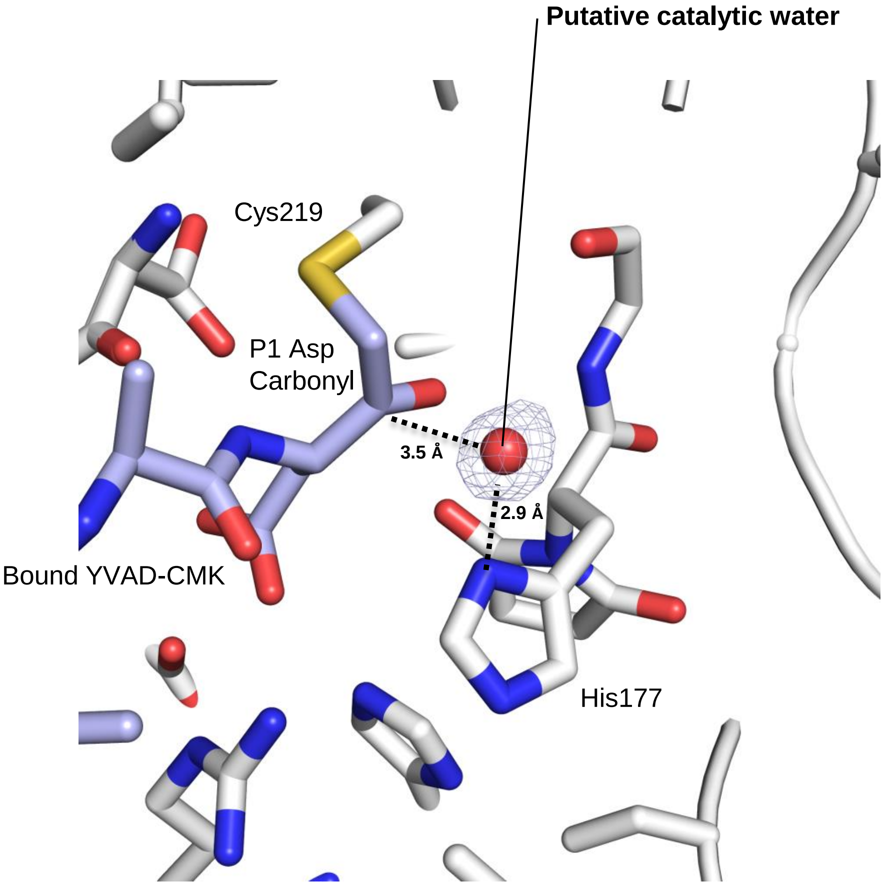

Supplementary Figure 3: Position of putative catalytic water.
The catalytic water is clearly visible within the electron density (2Fo-Fc map, 1σ), which is perfectly positioned between the catalytic histidine 177 and the carbonyl of the P1 Asp of the substrate (blue).

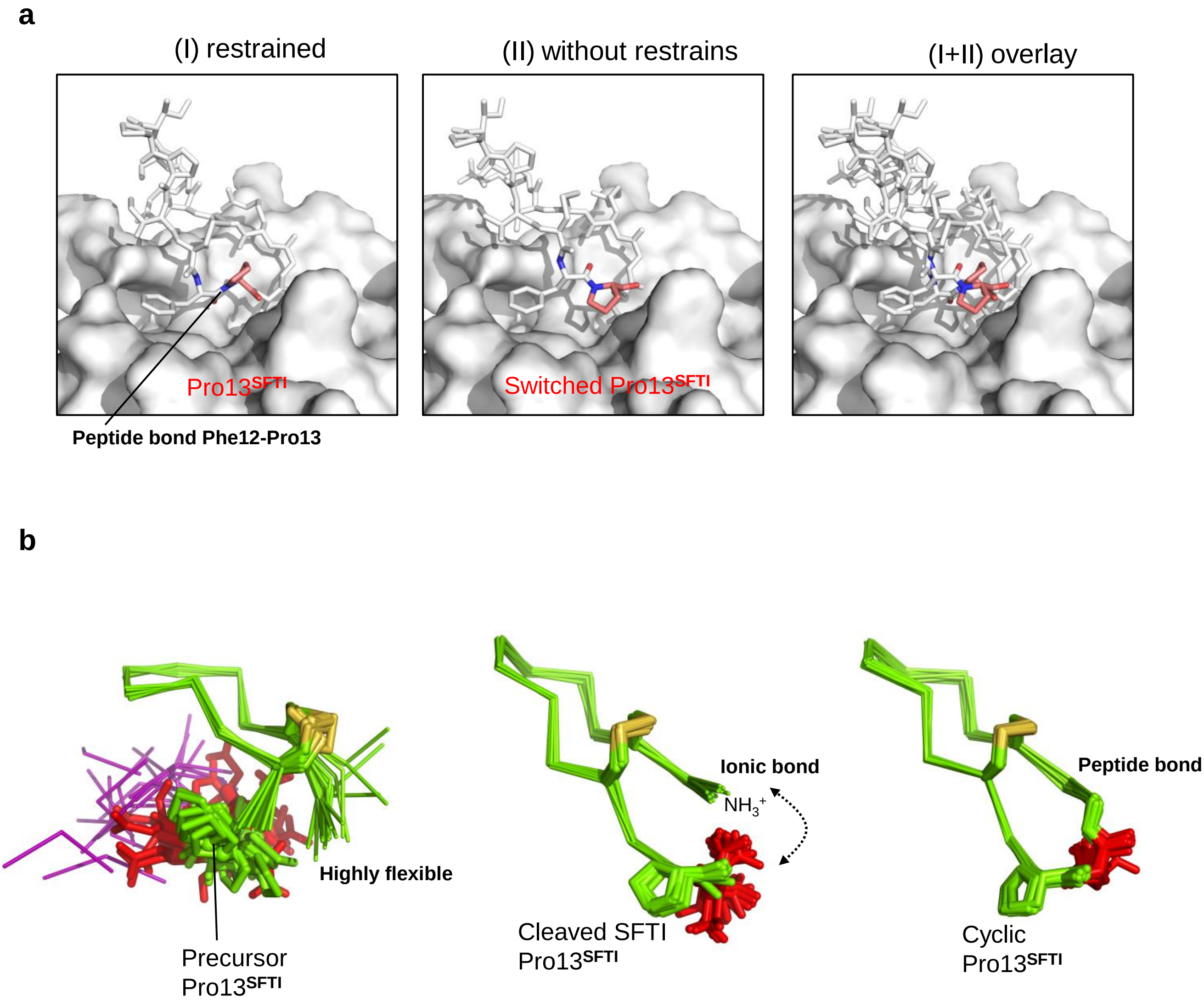

Supplementary Figure 4: Proline switch induced by cyclization.
(a) A docked cyclic SFTI is restrained to interact canonically (I). By releasing the restraints and energy optimization, Pro13 and the Pro13-Phe12 peptide bond is rotating (II). Thereby the Asp14 is pulled out of the S1 pocket and oxyanion hole. (I+II) Overlay of both structures shows the disruption of key interactions. (b) Within the precursor structure (NMR-ensemble, PDB-ID: 2ab9), the Asp14 and its primed residues, including Pro13, are highly flexible. Once a cyclic structure is formed (PDB-ID: 2jbl), Pro 13 is found in one restricted conformation, which is unfavorable for binding to AtLEGγ.

a

N-terminal albumin extension-N--GRCTRSIPPICFPD-GL---C-terminal albumin extension

b

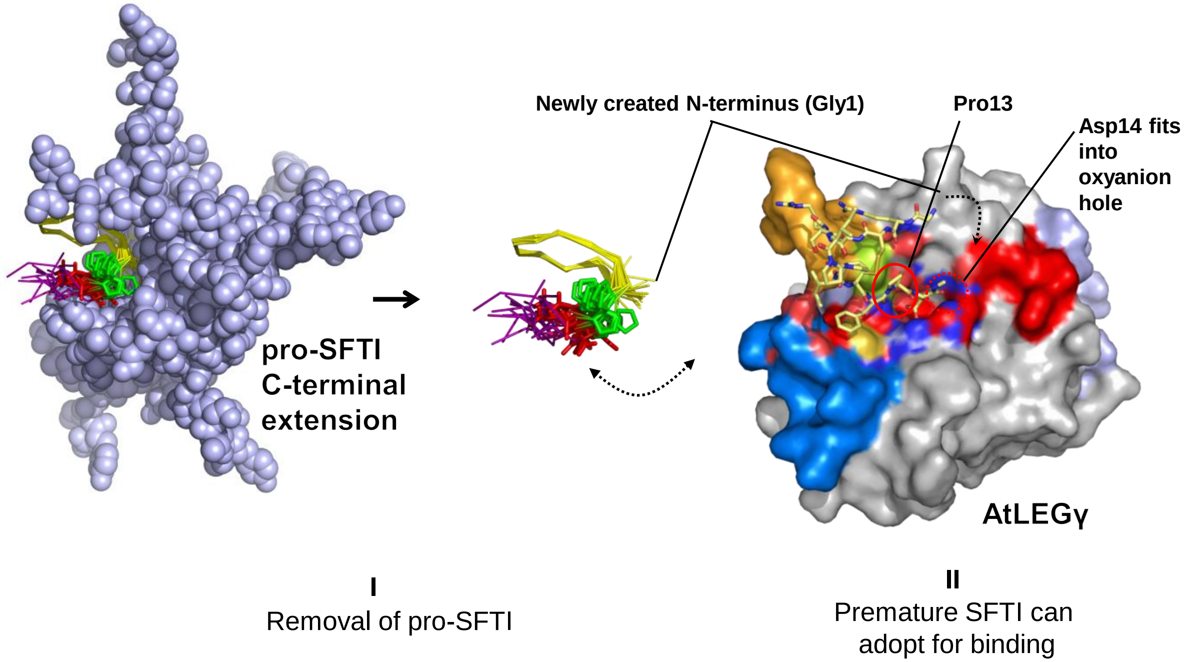

Supplementary Figure 5: Cleavage of the pro-peptide of SFTI allows binding to AtLEGγ.

(a) Schematic sequence of SFTI precursors. The N- and C-terminal albumin extensions are cleaved, at Asn (N) or Asp (D) residues, by legumain. (b) Representation of all NMR-structures of pro-SFTI (pdb entry 2AB9). Blue spheres represent the C-terminal pro-peptide of SFTI. Yellow ribbon: backbone of SFTI; Green sticks: Pro13; Red sticks: Asp14; purple ribbon, residues C-terminal of Asp14. The Propeptide of SFTI is removed by cleaving at a conserved asparagine, which precedes Gly1. This processing allows the flexible part (Asp14 and primed residues including Pro13) to adopt for canonical binding to AtLEGγ.

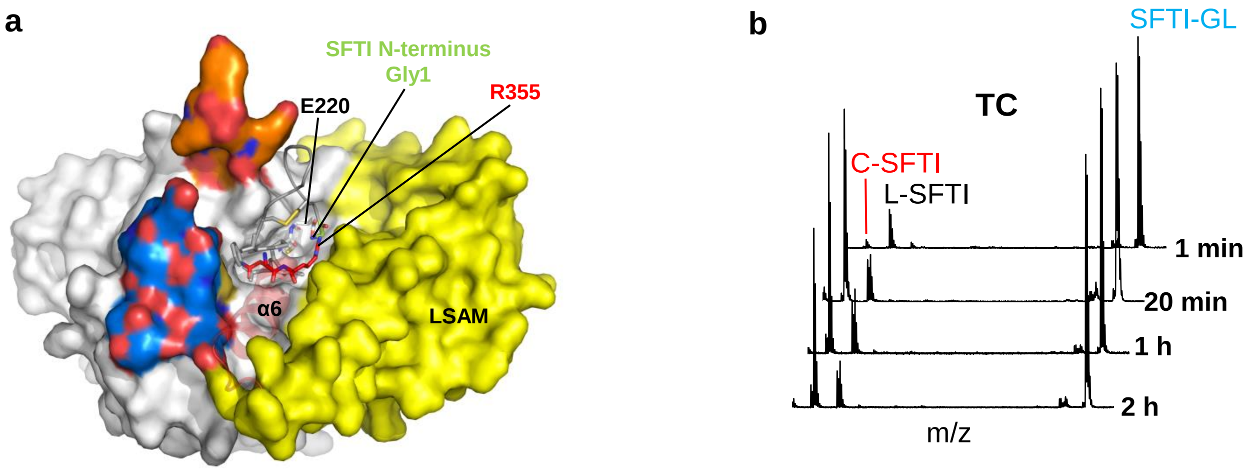

Supplementary Figure 6: Two chain state of AtLEGγ cyclizes SFTI.

(a) The two chain state of AtLEGγ is displayed in surface representation. c341, blue; c381, orange. The α6-helix is shown as transparent red cartoon. Please note the structural overlay of the SFTI N-terminus with the anchoring of the α6-helix by R355 to E220.

(b) Time-course experiment of SFTI-GL cyclization by two chain AtLEGγ at pH 6.5. SFTI-GL: unprocessed precursor, L-SFTI: processed (GL removed), C-SFTI: cyclised.

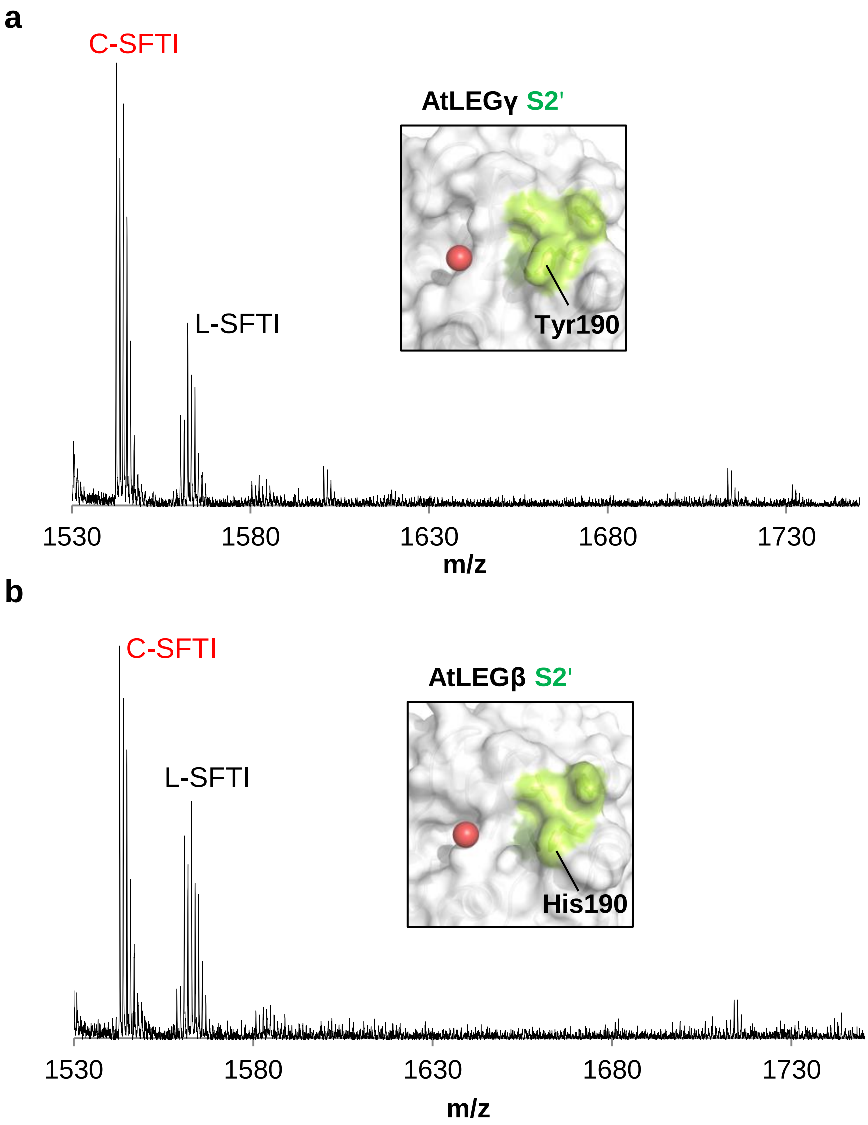

Supplementary Figure 7: AtLEGγ is a better cyclase than AtLEGβ. SFTI-GL was incubated with plant legumain for 12 h at 30°C at pH 6.0. (a) AtLEGγ mass spectra of cyclization experiment and its hydrophobic S2' pocket. (b) AtLEGβ MALDI-TOF spectra of cyclization experiment and its hydrophobic S2' pocket, which has a histidine at position 190, rendering it less hydrophobic. The crystal structure of AtLEGβ is unpublished data by Zauner et al..

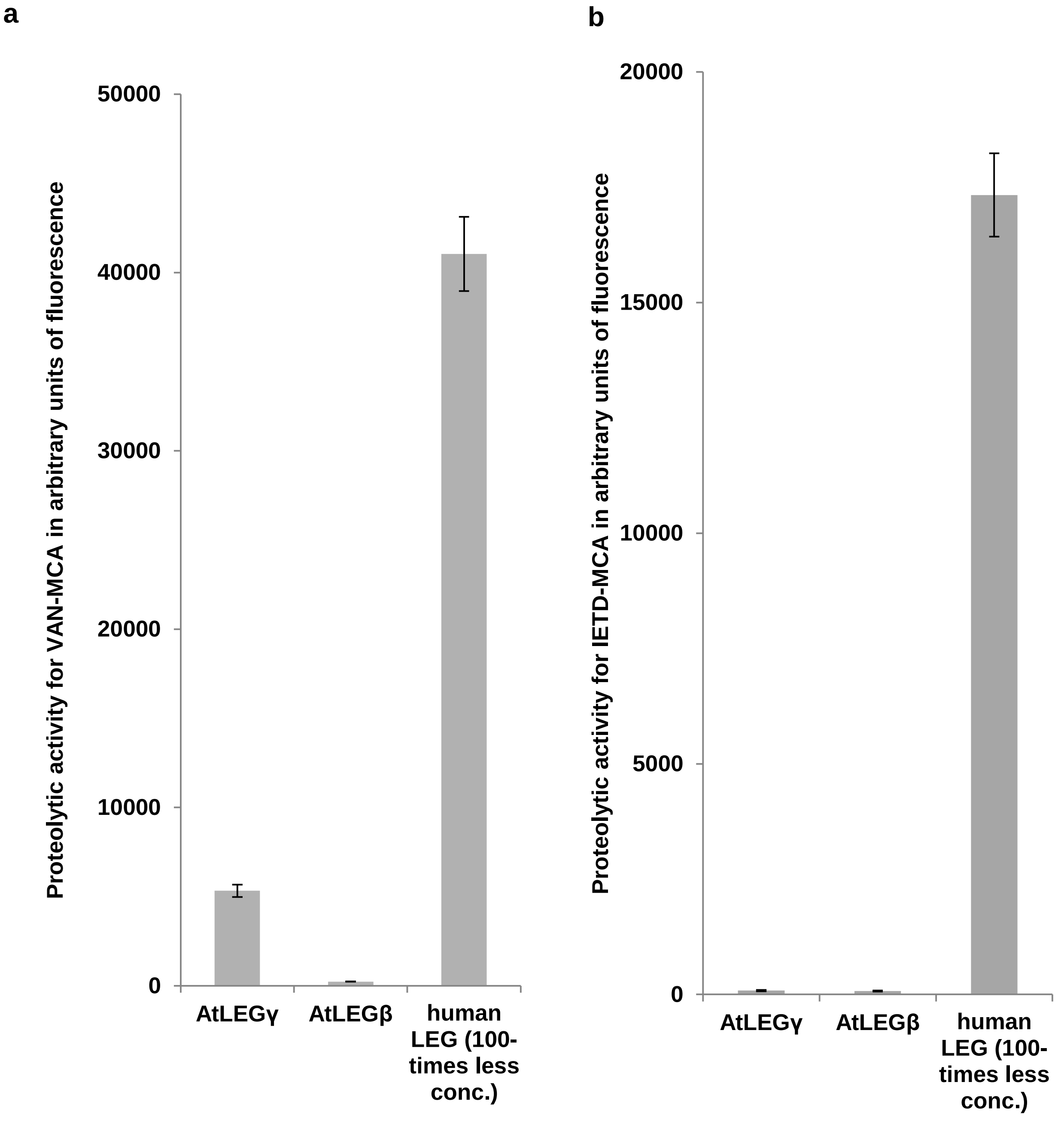

Supplementary Figure 8: Plant legumains show drastically lower hydrolytic activity.

Proteolytic activities of AtLEGγ and β and human legumain were compared at pH 4.0. (a) VAN-AMC substrate was used.(b) IETD-AMC substrate was used.

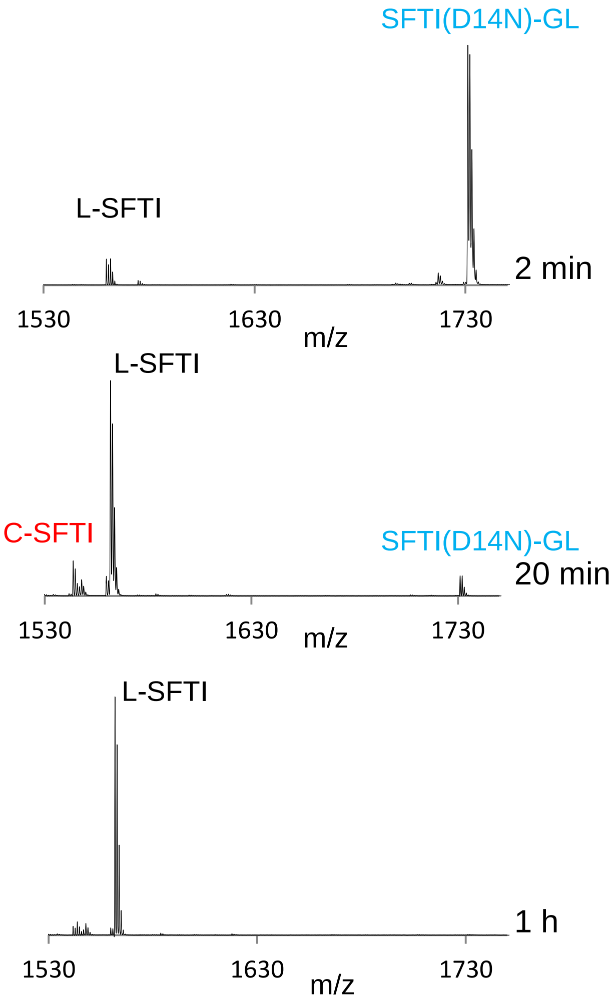

Supplementary Figure 9: D14N SFTI-GL is cyclized but rapidly cleaved again.
Mass spectra of a time-resolved ligation/transpeptidation experiment with SFTI(D14N)-GL. Please note that here the two chain state was used, since the speed of transpeptidation is slower, linear SFTI (L-SFTI; lacking the GL peptide) could be detected. After 20 min, cyclic SFTI (C-SFTI) can be detected which is cleaved again after further incubation (1 h).
